# Biological Reconstruction for Sarcoma

**DOI:** 10.1155/2013/648393

**Published:** 2013-08-05

**Authors:** Andreas Leithner, Per-Ulf Tunn, Pietro Ruggieri

**Affiliations:** ^1^Department of Orthopaedic Surgery, Medical University of Graz, 8036 Graz, Austria; ^2^Department of Orthopedic Oncology, Sarcoma Center Berlin-Brandenburg, Helios Klinikum Berlin-Buch, 13125 Berlin, Germany; ^3^Department of Orthopaedics, Orthopaedic Oncology Service, Istituto Ortopedico Rizzoli, University of Bologna, 40136 Bologna, Italy

Particularly in children and young adults, bone allografts and autografts seem to be a perfect and long-lasting alternative to the use of prostheses after bone resection due to bone or soft tissue sarcomas. However, several topics like osteoarticular massive allografts are still discussed controversially. Might allograft-prosthetic composites reconstructions serve as stable and durable alternatives? Questions like this as well as the long-term results have not been answered sufficiently yet. 

In a review, L. A. Holzer and A. Leithner focus on the historical highlights, the present role, and possible future options for biological extremity reconstruction. While hand transplantation is already an option, above-elbow transplantation or limb regeneration might become a topic in the future.

K. Rabitsch et al. report their experience with intercalary reconstructions of the lower limb using a vascularized fibula and an allograft in 12 patients. Although the event-free survival was only 51% at a two-year follow-up full weight bearing was achieved in all cases with all autografts still in place. The authors conclude that this method is a stable and durable reconstruction technique.

M. Niethard et al. illustrate their technique using bilateral vascularized fibular grafts for reconstruction of metadiaphyseal defects of the femur and tibia in 11 patients. Despite a similar complication rate as reported by K. Rabitsch et al., all of the complications were manageable without the loss of the biological reconstruction. The authors also report on the increased risk of fixation failure after radiotherapy.

L. E. Ritacco et al. describe the workflow for structural allograft selection of their renowned three-dimensional virtual bone bank system. Preoperative planning includes a 3D CT-derived bone model to define the exact size and shape in comparison to the planned resection. 

F. Traub et al. provide a retrospective analysis of their experience in biological reconstruction following the resection of pelvic tumors. Twenty-seven patients were evaluated for oncological as well as clinical and functional outcome. Hip transposition was used in 16 patients, and autologous nonvascularized fibular grafts were used in five patients. Despite the difficult situation after pelvic resection, MSTS score (mean 16.5) was good or excellent in most of the patients.

The second paper by K. Rabitsch et al. reports on the technique of distal radius osteoarticular allografts, which were used in five patients. With all allografts still in place at a mean follow-up of 32 months the functional results were good or excellent (DASH 8, Mayo wrist 84).

L. A. Aponte-Tinao et al. describe their high level of experience in biological upper extremity reconstruction in 70 patients, including 38 osteoarticular allografts, 24 allograft-prosthetic composites, and eight intercalary allografts. After a mean follow-up of 5 years the authors conclude that intercalary humeral allografts had the best outcome, while the other techniques led to articular deterioration, fracture, and allograft resorption with the need for revision surgeries in 16 patients.

G. L. Farfalli et al. compare 50 nonconstrained knee allograft-prosthetic composites with 36 matched constrained ones. In both groups the authors observed more allograft fractures when the prosthetic stem did not bypass the host-donor osteotomy. However, both groups had good or excellent MSTS functional scores. 

These papers represent an exciting and insightful snapshot of the current techniques of biological reconstruction after sarcoma resection. Some sophisticated methods, existing challenges, and possible future topics are highlighted in this special issue, which may inspire the reader and provide a stimulus for the present discussion on limb salvage techniques. 

